# Subjective Olfactory Impairment in a Patient Undergoing Anti-Obesity Pharmacotherapy: A Case of Symptom–Test Discrepancy

**DOI:** 10.3390/life15091349

**Published:** 2025-08-26

**Authors:** Hye Jun Lee, Hyun Jin Min

**Affiliations:** 1 Department of Family Medicine, College of Medicine, Chung-Ang University, 224-1 Heukseok-dong, Dongjak-gu, Seoul 06973, Republic of Korea; infiniteme@cau.ac.kr; 2 Department of Otorhinolaryngology-Head and Neck Surgery, College of Medicine, Chung-Ang University, 224-1 Heukseok-dong, Dongjak-gu, Seoul 06973, Republic of Korea

**Keywords:** anti-obesity medications, GLP-1 receptor agonists, semaglutide, olfactory dysfunction, subjective olfactory symptoms

## Abstract

With the growing use of anti-obesity medications (AOMs), particularly glucagon-like peptide-1 (GLP-1) receptor agonists, interest in their impact on chemosensory function has increased. We report a case of subjective olfactory discomfort that developed during AOM therapy despite normal objective test results. A 41-year-old woman received the GLP-1 receptor agonist semaglutide for 24 weeks. Thorough olfactory function evaluation was performed using the Questionnaire of Olfactory Disorders (a validated questionnaire), the Yonsei Olfactory Function Test (for psychophysical testing), and chemical gustometry. The tests were performed before treatment and after 24 weeks of AOM therapy. At baseline, the patient had no olfactory complaints and showed normal test results. After treatment, she reported significant subjective olfactory discomfort (visual analog scale score: 48, maximum: 50), with decreased quality of life and parosmia, despite stable Yonsei Olfactory Function scores and normal gustatory function. This case highlights the possible mismatch between subjective and objective olfactory assessment during AOM therapy. Comprehensive chemosensory evaluations and interdisciplinary collaboration are essential, and further large-scale long-term studies are required.

## 1. Introduction

The global prevalence of obesity has nearly tripled since 1975. According to the Global Burden of Disease Study, age-standardized rates had increased from approximately 4.6% to 14.0% by 2019, and over 890 million adults were affected in 2022. There has been a corresponding increase in obesity-related comorbidities, such as type 2 diabetes, dyslipidemia, cardiovascular disease, and non-alcoholic fatty liver disease [1]. This alarming epidemiological trend has prompted a more proactive approach to obesity management, including a marked increase in prescriptions for anti-obesity medications (AOMs), such as glucagon-like peptide-1 receptor agonists (GLP-1 RAs; including semaglutide). As the number of patients receiving pharmacological treatment for obesity continues to increase, emerging studies have begun to investigate the associations between AOMs and changes in olfactory function and broader chemosensory functions, building on a growing body of scientific evidence linking obesity with chemosensory alterations [2]. Several studies have reported that GLP-1 RAs could act through receptors present in the hippocampus and mitral cells in the olfactory bulb [3,4]. For example, in a case–control study comparing patients treated with GLP-1 RAs to matched controls, significant decreases in taste scores were reported across all five basic tastes (*p* < 0.001), whereas the smell scores showed a nonsignificant trend (*p* = 0.076) [5]. In addition, in a cohort of patients with obesity and type 2 diabetes, 3 months of GLP-1 RA therapy improved both olfactory test scores and odor-evoked brain activation in the parahippocampus, as well as yielding cognitive benefits [6].

Olfactory and gustatory functions are closely interconnected [7] and discrepancies frequently exist between subjective complaints of olfactory dysfunction and objective findings from clinical olfactory tests [8]. Therefore, a comprehensive evaluation of olfactory function should encompass both subjective and objective assessments and consider gustatory function as an integral component.

Given that patients undergoing AOM treatment typically visit non-otorhinolaryngology specialties, systematic evaluation of olfactory function is rarely performed in this population. Recently, we initiated a prospective cohort study involving patients receiving AOM therapy and conducted thorough assessments before and after treatment, including subjective and objective olfactory tests and gustatory function evaluations. In this case study, we focus on a patient who reported significant subjective olfactory discomfort despite having normal objective olfactory and gustatory test results following AOM treatment. We present this case to highlight the need for a multidimensional approach for chemosensory evaluation in patients treated with AOMs.

## 2. Detailed Case Description

A 41-year-old woman visited our hospital’s Family Medicine outpatient clinic for AOM treatment. She had a medical history of allergic rhinitis and benign breast cysts with no other significant internal medical conditions. On the first day of her visit, her anthropometric measurements were height 168 cm, weight 71 kg, body mass index 25.2 kg/m^2^, skeletal muscle mass 26.7 kg, body fat percentage 30.3%, and waist circumference 80.1 cm (Table 1). Anthropometric and body composition measurements were performed using bioelectrical impedance analysis (InBody 970; InBody Co., Ltd., Seoul, Republic of Korea). Venous blood samples for comprehensive laboratory evaluation were collected in the morning after a minimum of 8 h of overnight fasting. Biochemical analyses encompassed a wide range of metabolic and endocrine markers, including serum uric acid, fasting plasma glucose, liver enzymes such as aspartate aminotransferase, alanine aminotransferase, and gamma-glutamyl transferase, and fasting insulin levels. Additionally, lipid metabolism was assessed by measuring total cholesterol, triglycerides, high-density lipoprotein cholesterol, and low-density lipoprotein cholesterol levels. Systemic inflammation was evaluated using high-sensitivity C-reactive protein, and thyroid function was investigated by measuring free thyroxine and thyroid-stimulating hormone levels.

**Figure 1:**
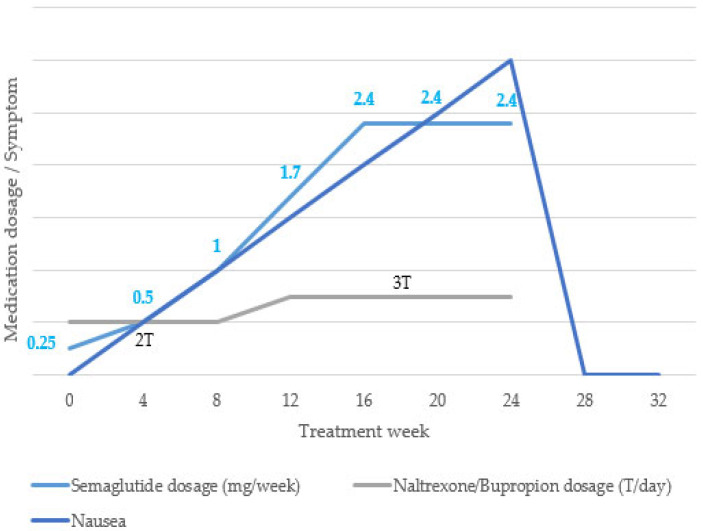
Treatment timeline showing anti-obesity medication dosage and nausea progression.

After reviewing the baseline test results, we decided to proceed with 24 weeks of pharmacological treatment with semaglutide and naltrexone/bupropion. During the 24-week treatment period, semaglutide was administered subcutaneously once a week. The dose was increased from 0.25 mg to 0.5 mg, 1 mg, 1.7 mg, and 2.4 mg every 4 weeks, and the maintenance dose (2.4 mg) was used up to 24 weeks. Naltrexone/bupropion 8/90 mg was administered twice daily (1T after breakfast and 1T after dinner) for the first 12 weeks and then escalated to twice daily (2T after breakfast and 1T after dinner) for the next 12 weeks (Figure 1).

Before the initiation and end of pharmacological treatment, the patient underwent a series of comprehensive sinus endoscopy and a clinical interview conducted by an experienced otolaryngologist, confirming the absence of sinusitis or allergic rhinitis at the time of evaluation. No findings suggestive of allergic rhinitis, such as watery rhinorrhea or pale mucosa were observed. The participants did not take any medications for sinusitis, allergic rhinitis, or related symptoms during the period of AOM. Validated chemosensory functional assessments, including the Questionnaire of Olfactory Disorders (QOD) to evaluate subjective olfactory discomfort [9], the Yonsei Olfactory Function Test (YOF) (RHICO Medical Co., Seoul, Republic of Korea), a psychophysical olfactory function test validated for the Korean population [10], and a validated chemical gustatory function test [11]. All assessment tools have demonstrated good reliability and validity in Korean populations. The QOD utilized in the study was conceptualized and developed by Hummel et al. in 2005 [12]. Since its initial development, the instrument has undergone cultural adaptation and psychometric validation to ensure its appropriateness and reliability for application in Korean populations. The QOD comprises four distinct domains designed to capture various dimensions of olfactory dysfunction: a parosmia-related statement (QOD-P), a life quality impact statement (QOD-LQ), a sincerity or reliability assessment statement (QOD-S), which allowed us to indirectly assess the participants’ psychological conditions, and a visual analog scale (QOD-VAS) reflecting subjective olfactory perception.

The YOF test evaluates three core domains of olfactory ability: detection threshold, odor discrimination, and odor identification. These components were combined to generate a total threshold discrimination identification (TDI) score with possible values ranging from 1 to 36. For the threshold assessment, 2-phenylethyl alcohol was used as the odorant, and the concentration levels were adjusted in response to the participants’ responses. The final threshold score was computed by averaging the last four of the seven reversals in the staircase paradigm. In the discrimination task, patients were presented with triplets of odor pens—two with identical odors and one with a different scent—and asked to identify the pen that smelled different. The number of correct responses in the 12 trials was recorded. Identification was assessed by presenting patients with one odor at a time and requiring them to choose the correct label from four options in a forced-choice format. The overall TDI score was obtained by summing the results from all three subtests, with scores ≤ 21.0 interpreted as indicative of hyposmia.

The chemical gustatory function test included 30 taste solutions representing five basic taste qualities, sweet (sucrose), bitter (quinine hydrochloride), salty (sodium chloride), sour (citric acid), and umami (monosodium glutamate), each presented at six different concentrations. Recognition thresholds were evaluated by asking participants to select one of six options (“sweet,” “bitter,” “salty,” “sour,” “umami,” or “no taste”) to best describe each sample. The total gustatory function score ranged from 0 to 30 and was calculated by summing the correctly identified taste responses. A recognition threshold score < 12 was classified as hypogeusia. To ensure consistency, all assessments were performed by a single trained physician in a well-ventilated clinical laboratory setting.

In the presented case, the patient’s baseline scores were as follows: the sum of the raw QOD-LQ score (maximum score, 57) was 5, which transformed to an LQ score of 8.7 (LQ = raw LQ score/0.57 [%]); the sum of the QOD-S score (maximum score, 18) was 9, which converted to an S score of 1.62 (S = raw S score/0.18 [%]); and the sum of the raw QOD-P score (maximum score, 12) was 6, which converted to a P score of 50 (*p* = raw P score/0.12 [%]) [9]. The sum of the overall olfactory discomfort score, which was the raw QOD-VAS score (maximum score, 50), was 0. The YOF had a threshold score of 4, discrimination score of 9, and identification score of 12, with a total TDI score of 25, indicating normosmia [10]. Chemical gustometry revealed a detection threshold of 6 and recognition threshold of 24, consistent with normal gustatory function [11].

Toward the end of the pharmacological treatment period, the patient began to experience olfactory disturbances, which led to significant discomfort in daily life. After 24 weeks of treatment, the patient’s anthropometric measurements showed improvement, with their height of 168 cm and weight of 67 kg resulting in a body mass index of 23.7 kg/m^2^. At this time, their follow-up QOD-LQ score was 11, their converted LQ score was 19.25, their QOD-S score was 6, their converted S score was 1.08, their QOD-P score was 8, and their converted P score was 66. Their overall QOD-VAS score was 48. However, the YOF test showed a threshold score of 4, discrimination score of 11, and identification score of 12, with a total TDI score of 27, indicating normosmia [10]. Moreover, chemical gustometry showed a detection threshold of 6 and a recognition threshold of 23, which were consistent with normal gustatory function [11]. Two months after cessation of AOM, the patient’s olfactory function and general physical condition had returned to normal, with no sequelae or residual discomfort reported.

## 3. Discussion

As the number of patients receiving AOMs, such as GLP-1 RAs, continues to increase, recent studies have begun to explore the potential therapeutic effects of GLP-1 RAs on psychological behavior and olfactory networks, building upon earlier findings linking obesity and olfaction [13]. For instance, patients with both obesity and diabetes exhibit lower general cognitive performance and reduced olfactory threshold scores, accompanied by decreased activation in the left hippocampus and altered seed-based functional connectivity with the right insula, compared to diabetic patients without obesity. Notably, after 3 months of treatment with GLP-1 RAs, these patients demonstrated significant improvements in their Montreal Cognitive Assessment scores and total olfactory test performance, and enhanced odor-induced activation in the right parahippocampal region [6]. However, a thorough understanding of the olfactory system and a systematic evaluation protocol are required to accurately investigate the relationship between AOM and olfactory function. In most clinical settings, the departments prescribing AOM and those conducting olfactory assessments are separate, making it challenging to integrate comprehensive evaluations. Studies including in-depth assessments of subjective olfactory complaints, psychophysical olfactory testing, and gustatory function tests are therefore extremely rare. To overcome this limitation, we conducted a prospective, comprehensive evaluation of olfactory function in patients undergoing anti-obesity pharmacotherapy. It included a validated Korean version of a questionnaire assessing subjective olfactory discomfort, psychophysical olfactory testing, and chemical gustatory function testing, all of which demonstrated diagnostic utility in the Korean population. Consequently, we encountered the case described in this report. The patient did not report any subjective olfactory symptoms and showed normal results in both olfactory and gustatory tests before initiating AOM. However, by week 24 of GLP-1 RA treatment, the patient had developed clear subjective olfactory complaints despite showing no detectable abnormalities in objective testing. Notably, there were significant declines in their QOD-LQ, QOD-P, and QOD-VAS scores, suggesting the deterioration of olfaction-related quality of life and the emergence of parosmia symptoms; a QOD-VAS score of 48 out of 50 indicated that the discomfort had reached a maximal level. The psychophysical olfactory function tests that are commonly used for diagnostic purposes do not capture the impact of olfactory symptoms on patients’ quality of life, or the presence of qualitative disturbances such as parosmia.

Semaglutide, a widely used GLP-1 RA, exerts therapeutic effects by enhancing insulin secretion in hyperglycemic states, inhibiting glucagon release under hyperglycemic or euglycemic conditions, delaying gastric emptying, and reducing food intake [14]. However, this medication is known to cause a range of adverse gastrointestinal effects, including nausea, vomiting, diarrhea, constipation, and abdominal pain. Naltrexone/bupropion, another AOM commonly used in combination therapy, stimulates pro-opiomelanocortin neurons to suppress appetite; its common side effects include nausea, headache, constipation, and dizziness [15]. Based on pharmacokinetic profiles, the time to reach maximum plasma concentration (T_max_) is approximately 1–3 d for semaglutide [16], about 1 h for naltrexone, and approximately 3 h for bupropion [17]. The elimination half-lives are approximately 1 week (165 h) for semaglutide [16], 4 h for naltrexone, and 21 h for bupropion [17]. Although dose escalation is associated with an increased incidence of adverse effects, it has been reported that adverse events with naltrexone/bupropion tend to occur earlier, primarily within the first 1–2 weeks, compared to semaglutide [17]. In the present case, the patient reported olfactory dysfunction accompanied by discomfort in daily activities toward the end of a 24-week course of AOM. Given that the dose of naltrexone/bupropion was maintained after 12 weeks, while the dose of semaglutide was increased until 20 weeks, it is plausible to hypothesize that semaglutide played a primary role in inducing nausea, which may have contributed to the subjective olfactory discomfort. However, given the overlapping period of central nervous system adaptation and the pharmacodynamic profiles of both agents, it is important to consider potential combined effects. The extended titration period for semaglutide suggests a stronger temporal relationship with symptom onset.

A previous study reported that semaglutide induced hyperemesis gravidarum in pregnant women [18]. In actual clinical practice, especially when semaglutide was used, the nausea that prompted patient complaints showed symptoms similar to morning sickness and hyperemesis gravidarum (HG) in its severe form. Moreover, one study that showed that the median for correct smell identification in HG patients showed significantly lower olfactory dysfunction compared to the control group [19], and another three-arm study reported that HG women performed the worst in smell identification [20]. However, large-scale long-term studies are required to confirm this causal relationship. As there is a lack of studies evaluating both subjective and objective olfactory function at different time points in specific AOM treatment regimes, further prospective studies with larger sample sizes are warranted to clarify this causal relationship. Conversely, the olfactory discomfort caused by AOM may induce nausea. Olfactory stimuli often cause nausea, vomiting, hyperemesis of the gravidarum, and migraines. One study reported that the nausea and vomiting experienced by women with hyperemesis gravidarum share a common mechanism with migraine headaches, possibly based on allelic variations within the dopaminergic receptor (DRD2) gene associated with olfaction [21]. Therefore, it is also possible that subjective olfactory discomfort caused the nausea.

A major limitation of this study is its single-case design, which restricts the generalizability of the findings. Consequently, the study does not allow for a thorough investigation into the mechanisms underlying the discrepancy between objective and subjective olfactory assessments or mechanisms underlying changes in sensory processing in the central nervous system and alterations in the threshold of olfactory sensitivity following anti-obesity medication. Previous animal studies have shown that diet-induced obesity leads to structural degeneration of the olfactory sensory neurons [22]. Hormonal signaling via leptin, insulin, and ghrelin receptors in the olfactory bulb and mucosa also influences odor detection and processing, and such hormonal imbalances are common in obesity [23]. Therefore, the potential effects of GLP-1 receptor agonists on central sensory processing should be investigated in future studies. Moreover, comparative analysis between different types of AOMs and GLP-1 Ras was not feasible in this study. Olfactory dysfunction is commonly observed in patients with diabetes, and previous studies on GLP-1 Ras, such as exenatide and liraglutide, have reported improvements in olfactory function [24]. In contrast, the present study observed subjective olfactory impairment following semaglutide treatment, underscoring a need for further research to understand the discrepancy between objective and subjective olfactory assessments, as well as the different types of GLP-1 RAs.

## 4. Conclusions

In conclusion, this case study demonstrates that a comprehensive evaluation, including validated subjective questionnaires that address symptoms, such as parosmia, is essential to assess the true olfactory impact of AOM. We aimed to share this insight and emphasize the importance of interdisciplinary collaboration between clinical departments prescribing AOM and otolaryngology departments capable of conducting systematic olfactory assessments. Such collaboration is crucial for producing more accurate and meaningful findings for future research. Moreover, a comprehensive evaluation of olfactory function should encompass both subjective and objective assessments. Furthermore, long-term follow-up observational studies with large populations are warranted to evaluate changes in olfactory function associated with the use of AOMs, such as GLP-1 RAs.

## Figures and Tables

**Table 1:** Comparison of patient’s clinical features before and after anti-obesity treatment.

Measurement	Before Anti-Obesity Medication	After Anti-Obesity Medication
**Anthropometric Measurements**		
Height (cm)	168	168
Body weight (kg)	71	67
Body mass index (kg/m^2^)	25.2	23.7
Waist circumference (cm)	80.1	-
**Blood test**		
fasting glucose (mg/dL)	92	89
hs-CRP (mg/L)	1.32	-
AST (IU/L)	22	27
ALT (IU/L)	19	44
Total cholesterol (mg/dL)	198	186
Triglyceride (mg/dL)	117	140
HDL-cholesterol (mg/dL)	38	27
LDL-cholesterol (mg/dL)	127	125
free T4 (ng/dL)	1.32	-
TSH (μIU/mL)	4.17	-
**QOD test**		
QOD-LQ (LQ)	5 (8.77)	11 (19.30)
QOD-S (S)	9 (1.62)	6 (1.08)
QOD-P (P)	6 (50)	8 (66.67)
QOD-VAS	0	48
**YOF test**		
Threshold	4	4
Discrimination	9	11
Identification	12	12
TDI	25	27
**Chemical gustatory function test**		
Detection threshold	6	6
Recognition threshold	24	23

Abbreviations: hs-CRP, high-sensitivity C-reactive protein; AST, aspartate aminotransferase; ALT, alanine aminotransferase; HDL-cholesterol, high-density lipoprotein cholesterol; LDL-cholesterol, low-density lipoprotein cholesterol; free T4, free thyroxine; TDI, threshold discrimination identification score; TSH, thyroid-stimulating hormone; QOD test, Questionnaire of Olfactory Disorders test; QOD-LQ, QOD-life quality; QOD-S, QOD-sincerity; QOD-P, QOD-parosmia; QOD-VAS, QOD-visual analog scale; YOF test, Yonsei Olfactory Function Test.

## Data Availability

The datasets generated and analyzed in the current study are not publicly available because of the privacy of the enrolled patients; however, they are available from the corresponding author upon reasonable request.

## Abbreviations

The following abbreviations are used in this manuscript:

AOM	Anti-obesity medication
GLP-1 RA	Glucagon-like peptide-1 receptor agonist
HG	Hyperemesis gravidarum
QOD	Questionnaire of olfactory disorders
QOD-P	Parosmia-related statement of the QOD
QOD-LQ	Life quality impact statement of the QOD
QOD-S	Sincerity or reliability assessment statement of the QOD
QOD-VAS	Visual analog scale of the QOD
TDI	Threshold discrimination identification
YOF	Yonsei olfactory function test
